# Higher glycemic variability within the first day of ICU admission is associated with increased 30-day mortality in ICU patients with sepsis

**DOI:** 10.1186/s13613-020-0635-3

**Published:** 2020-02-07

**Authors:** Wen-Cheng Chao, Chien-Hua Tseng, Chieh-Liang Wu, Sou-Jen Shih, Chi-Yuan Yi, Ming-Cheng Chan

**Affiliations:** 1 Division of Chest Medicine, Department of Internal Medicine, Taichung Veterans General Hospital, 1650 Taiwan Boulevard Sect. 4, Taichung, 40705 Taiwan; 2 Department of Critical Care Medicine, Taichung Veterans General Hospital, 1650 Taiwan Boulevard Sect. 4, Taichung, 40705 Taiwan; 3 Division of Pulmonary Medicine, Department of Internal Medicine, Shuang Ho Hospital, Taipei Medical University, Taipei, Taiwan; 4 Division of Pulmonary Medicine, Department of Internal Medicine, School of Medicine, College of Medicine, Taipei Medical University, Taipei, Taiwan; 5 Center of Quality Management, Taichung Veterans General Hospital, 1650 Taiwan Boulevard Sect. 4, Taichung, 40705 Taiwan; 6 Department of Automatic Control Engineering, Feng Chia University, Taichung, Taiwan; 7 Department of Nursing, Taichung Veterans General Hospital, 1650 Taiwan Boulevard Sect. 4, Taichung, 40705 Taiwan; 8 Division of Critical Care and Respiratory Therapy, Department of Internal Medicine, Taichung Veterans General Hospital, 1650 Taiwan Boulevard Sect. 4, Taichung, 40705 Taiwan; 9 Central Taiwan University of Science and Technology, Taichung, Taiwan; 10 The College of Science, Tunghai University, Taichung, 40704 Taiwan

**Keywords:** Glycemic variability, Glycemic control, Sepsis

## Abstract

**Background:**

High glycemic variability (GV) is common in critically ill patients; however, the prevalence and mortality association with early GV in patients with sepsis remains unclear.

**Methods:**

This retrospective cohort study was conducted in a medical intensive care unit (ICU) in central Taiwan. Patients in the ICU with sepsis between January 2014 and December 2015 were included for analysis. All of these patients received protocol-based management, including blood sugar monitoring every 2 h for the first 24 h of ICU admission. Mean amplitude of glycemic excursions (MAGE) and coefficient of variation (CoV) were used to assess GV.

**Results:**

A total of 452 patients (mean age 71.4 ± 14.7 years; 76.7% men) were enrolled for analysis. They were divided into high GV (43.4%, 196/452) and low GV (56.6%, 256/512) groups using MAGE 65 mg/dL as the cut-off point. Patients with high GV tended to have higher HbA1c (6.7 ± 1.8% vs. 5.9 ± 0.9%, *p* < 0.01) and were more likely to have diabetes mellitus (DM) (50.0% vs. 23.4%, *p* < 0.01) compared with those in the low GV group. Kaplan–Meier analysis showed that a high GV was associated with increased 30-day mortality (log-rank test, *p* = 0.018). The association remained strong in the non-DM (log-rank test, *p* = 0.035), but not in the DM (log-rank test, *p* = 0.254) group. Multivariate Cox proportional hazard regression analysis identified that high APACHE II score (adjusted hazard ratio (aHR) 1.045, 95% confidence interval (CI) 1.013–1.078), high serum lactate level at 0 h (aHR 1.009, 95% CI 1.003–1.014), having chronic airway disease (aHR 0.478, 95% CI 0.302–0.756), high mean day 1 glucose (aHR 1.008, 95% CI 1.000–1.016), and high MAGE (aHR 1.607, 95% CI 1.008–2.563) were independently associated with increased 30-day mortality. The association with 30-day mortality remained consistent when using CoV to assess GV.

**Conclusions:**

We found that approximately 40% of the septic patients had a high early GV, defined as MAGE > 65 mg/dL. Higher GV within 24 h of ICU admission was independently associated with increased 30-day mortality. These findings highlight the need to monitor GV in septic patients early during an ICU admission.

## Background

Sepsis is one of the leading causes of death worldwide and the most common cause of death in patients admitted to an intensive care unit (ICU) [1]. Dysglycemia and optimal glycemic control remain important prognostic factors in patients with sepsis [2, 3]. Glycemic variability (GV) has recently been reported to be the third domain of sepsis-induced dysglycemia in addition to hyperglycemia and hypoglycemia [4]. However, there is no universal standard for how best to determine GV in patients with sepsis, particularly the number and timing of blood sugar samples required [5]. Previous studies have reported that a wide range of blood sugar samples are needed to calculate GV, ranging from 1 to 5 times/day [6, 7]. Moreover, a previous study using a continuous glucose monitoring system reported that a relatively small number of blood sugar samples in critically ill patients may underestimate GV [8]. When to determine GV in patients with sepsis is another important issue. GV in the early phase of sepsis may reflect a physiological response to stress; whereas in the later phase it may be affected by a variety of factors related to management, including nutritional intake and glucose control strategy [9, 10]. Previous studies have shown an association between mortality and GV in general ICU patients and selected ICU patients, such as those with diabetes [11–13]. However, few studies have focused on GV in the first 24 h of ICU admission due to sepsis, and thus the association between mortality and early GV in sepsis warrants further investigations. Therefore, we conducted this study with retrospective analysis of patients with sepsis who received protocol-based management with blood sugar monitoring every 2 h after ICU admission. The aim of this study was to investigate the prevalence of high GV and to determine the association between GV and mortality.

## Methods

### Subjects and data collection

This retrospective cohort study was conducted at one 24-bed medical ICU of a tertiary-care referral hospital with 1514 beds in central Taiwan. Databases of the sepsis management registry and electronic medical records which were collected prospectively were used for analysis. We retrospectively screened all of the adult patients listed in the sepsis management registry database between 1 January 2014 and 31 December 2015. In total, 517 consecutive patients who were admitted to the ICU with sepsis and received protocolized bundle care were included. The bundle care included antibiotic administration, pathogen identification and culture, lactate measurement, fluid resuscitation and vasopressors to stabilize hemodynamics. A protective ventilator strategy was used for the patients with respiratory failure needing mechanical ventilation by targeting tidal volume at 6 ml/kg and limiting the plateau pressure to less than 30 cm H_2_O. Glycemic control aiming at keeping blood sugar levels between 150 and 180 mg/dL was achieved using protocolized continuous insulin infusion. Blood glucose was monitored every 2 h for the first 24 h of ICU admission. Given that arterial catheter insertion is part of standard care for patients with sepsis, we used arterial blood instead of capillary blood for point-of-care testing using a glucose meter. The sepsis management registry and electronic medical records databases were used to obtain critical care-associated data, including demographics, comorbidities, Acute Physiology and Chronic Health Evaluation (APACHE) II score, serum glycated hemoglobin (HbA1c), serial glucose data, and other relevant data.

### Definition of DM and determination of glycemic variability

Diabetes mellitus (DM) in this study was defined as patients with a diagnosis of DM before admission and those with HbA1c ≥ 6.5% at admission even without a history of DM [14]. We used two measures to assess GV: mean amplitude of glycemic excursions (MAGE) and coefficient of variation (CoV). Briefly, MAGE is the mean blood glucose value exceeding the standard deviation (SD) from the 24-h mean blood glucose level [15], and CoV represents the ratio of the SD to the mean glucose level [16]. We used MAGE 65 mg/dL as the cut-off point given that a normal MAGE has been reported to be approximately less than 65 mg/dL [15, 17], and a CoV of 30% based on a previous study exploring dysglycemia in patients with sepsis [18].

### Statistical analysis

Data were presented as frequencies (percentages) for categorical variables and as means ± SDs for continuous variables. The Kolmogorov–Smirnov test was used to test normality. Differences between the two groups were analyzed using the Student’s *t* test or Mann–Whitney *U* test, while the Chi-square test with Fisher’s exact test were used for categorical variables. Kaplan–Meier analysis was used to test the association between 30-day mortality and GV using MAGE 65 mg/dL as the cut-off point. Variables were considered as candidates for inclusion in the multivariate model if the associated univariate *p* value was < 0.20, and variables which have been reported to associate with mortality in critically ill patients were also included [19]. A Cox proportional hazards regression model, adjusted for glycemia-associated variables and 30-day mortality-associated variables, was constructed to identify independent variables that predicted 30-day mortality. GV was determined using EasyGV Version 9.0.R2 software. Statistical significance was set at a two-sided *p* value of < 0.05. All data were analyzed using SPSS software version 22.0 (SPSS Inc., Chicago, IL, USA).

## Results

### Demographic and GV-related data

**Fig. 1:**
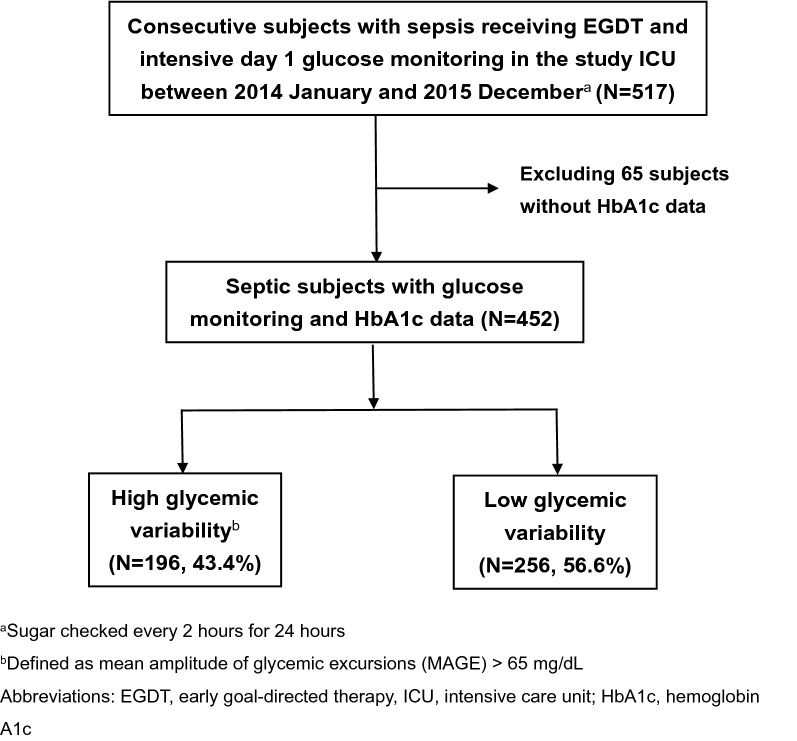
Flowchart of patient enrollment

A total of 517 consecutive patients were admitted to the medical ICU due to sepsis between January 2014 and December 2015, of whom 65 were excluded due to a lack of HbA1c data within the past 3 months (Fig. 1). Patients who died within a few hours after ICU admission were hence excluded given that HbA1C was generally checked on day 2 in the study ICU. The remaining 452 patients were eligible for analysis and were divided into high GV (*n* = 196, 43.4%) and low GV (*n* = 256, 56.6%) groups using MAGE 65 mg/dL as the cut-off point.

Table 1 summarizes the demographic, GV, and sepsis-related data (Table 1). The mean age was 71.4 ± 14.7 years, and 76.7% of the patients were male. The mean MAGE and CoV were 67 ± 51.1 mg/dL and 23.5 ± 11.2%, respectively. The most common underlying comorbidities were congestive heart failure (31.6%), chronic airway disease (28.1%) and malignancy (23.9%). As expected, those with high GV had a higher HbA1c (6.7 ± 1.8% vs. 5.9 ± 0.9%, *p* < 0.01) and were more likely to have DM (50.0% vs. 23.4%, *p* < 0.01) compared with those in the low GV group. The other variables appeared to be comparable between these two groups, except that those with high GV were less likely to be male (71.4% vs. 80.8%, *p* = 0.03) compared to those in the low GV group. These data suggested that a high GV was prevalent in the patients with sepsis and that it was associated with DM and levels of HbA1c.

**Table 1:** Characteristics of the 452 patients with sepsis categorized by glycemia variability

	All	Low GV	High CV	*p* value
		(MAGE ≦ 65)	(MAGE > 65)	
	(*N* = 452)	(*N* = 256)	(*N* = 196)	
Basic and glycemia data				
Age (years)	71.4 ± 14.7	71.7 ± 15.3	71.2 ± 13.9	0.72
Male %	346 (76.7%)	206 (80.8%)	140 (71.4%)	0.03
BMI (kg)	23.7 ± 8.8	24 ± 11	23.3 ± 4.5	0.36
HbA1c (%)	6.3 ± 1.4	5.9 ± 0.9	6.7 ± 1.8	< 0.01
Day 1 glucose metrics				
Mean glucose (mg/dL)	164.1 ± 41.7	148.7 ± 31.0	184.2 ± 45.1	< 0.01
Peak glucose (mg/Dl)	239.9 ± 81.6	194.7 ± 50.3	298.9 ± 76.9	< 0.01
Hypoglycemia (< 40 mg/dL)	2 (0.4%)	0 (0%)	2 (1.0%)	0.18
Glycemic variation				
MAGE	67 ± 51.1	33.7 ± 18.6	110.5 ± 47.2	< 0.01
CoV	23.5 ± 11.2	17.2 ± 7.1	31.8 ± 10.1	< 0.01
Comorbidities				
Diabetes mellitus	158 (35%)	60 (23.4%)	98 (50.0%)	< 0.01
Congestive heart failure	143 (31.6%)	74 (28.9%)	69 (35.2%)	0.19
Cerebrovascular disease	50 (11.1%)	27 (10.5%)	23 (11.7%)	0.80
Chronic airway disease	127 (28.1%)	70 (27.3%)	57 (29.1%)	0.76
Chronic renal disease	53 (11.7%)	26 (10.2%)	27 (13.8%)	0.30
Malignancy	108 (23.9%)	64 (25.0%)	44 22.4(%)	0.60
Severity-associated variables				
APACHE II score	27.4 ± 6.6	27.3 ± 6.6	27.5 ± 6.6	0.70
Lactate level, 0 h (mg/dl)	26.5 ± 23.6	24.5 ± 20.1	29.1 ± 27.4	0.05
Lactate level, 24 h (mg/dl)	21.7 ± 19.6	19.9 ± 15.9	24.2 ± 23.6	0.10
ScvO2, 0 h (%)	74.7 ± 11.4	74.4 ± 11.3	75.2 ± 11.7	0.45
ScvO2, 6 h (%)	74.6 ± 10.5	74.8 ± 10.5	74.3 ± 10.4	0.61
Laboratory data				
Albumin (mg/dL)	2.8 ± 0.6	2.9 ± 0.6	2.8 ± 0.6	0.21
Hemoglobin (g/dL)	10.2 ± 2.3	10.3 ± 2.3	10.1 ± 2.4	0.29
Creatinine (mg/dL)	2.1 ± 2.2	1.9 ± 1.8	2.3 ± 2.6	0.07
C-reactive protein (mg/dL)	13.8 ± 10.6	14 ± 10.2	13.7 ± 11.1	0.80
Procalcitonin (ng/mL)	17.4 ± 34.9	17 ± 34.5	17.8 ± 35.5	0.82
Outcomes				
30-day mortality	140 (31%)	68 (26.6%)	72 (36.7%)	0.03

Data are presented as mean ± SD and N (%)

*GV* glycemic variability, *MAGE* mean amplitude of glycemic excursions, *CoV* coefficient of variation, *DM* diabetes mellitus, *BMI* body-mass index, *HbA1c* hemoglobin A1c, *APACHE II* acute physiology and chronic health evaluation II, *ScvO2* central venous oxygen saturation

### High day 1 GV was associated with high 30-day mortality

**Fig. 2:**
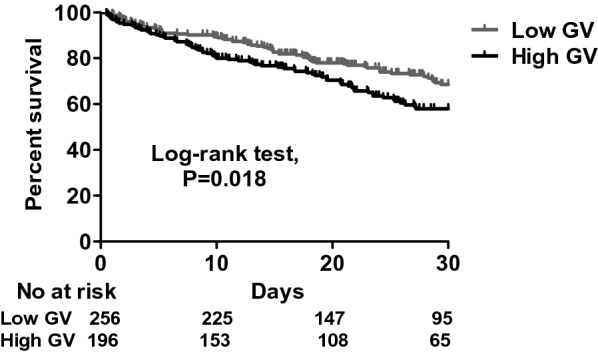
Kaplan–Meier survival curves categorized by glycemic variability. Low GV, MAGE ≤ 65; high GV, MAGE > 65

**Fig. 3:**
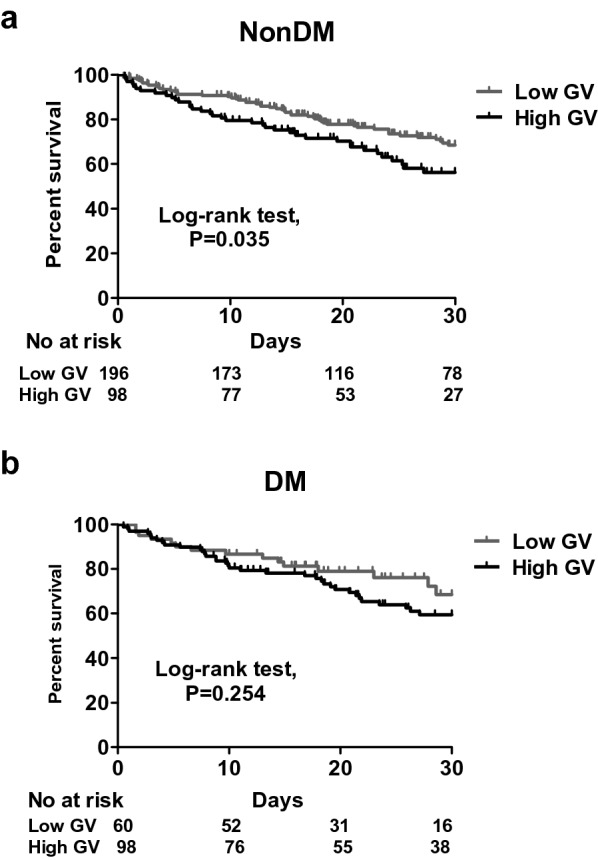
Kaplan–Meier survival curves categorized by glycemic status in patients without (**a**) and with (**b**) DM. Low GV, MAGE ≤ 65; high GV, MAGE > 65

**Fig. 4:**
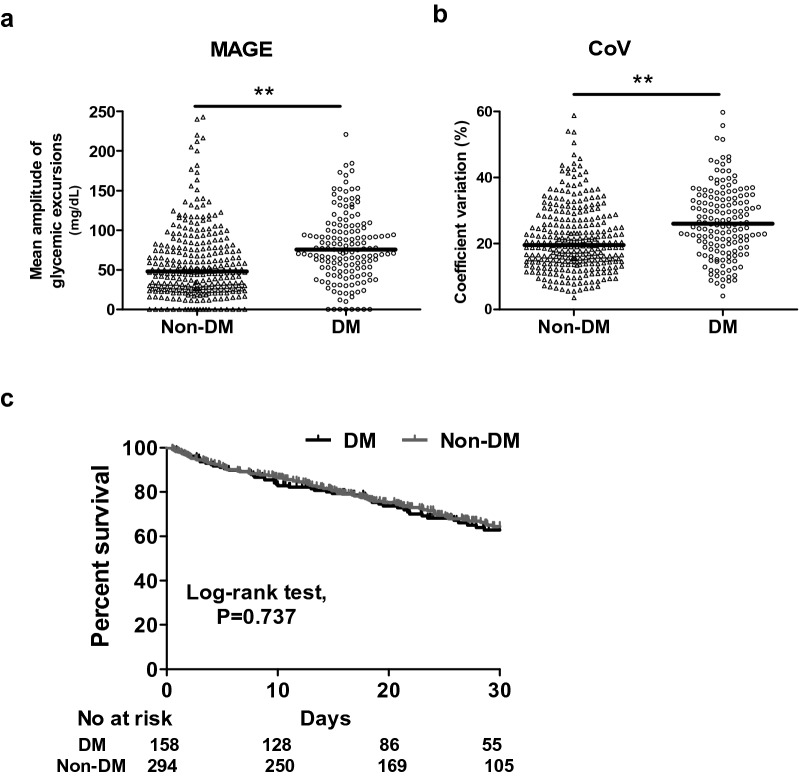
Individual glycemic variability (**a**, MAGE; **b**, CoV) in septic patients without and with DM. (C) Kaplan–Meier survival curves categorized by DM. **p < 0.005

The patients with high GV had a higher mean glucose level (184.2 ± 45.1 vs. 148.7 ± 31.0 mg/dL, *p* < 0.01) and peak glucose level (298.9 ± 76.9 vs. 194.7 ± 50.3 mg/dL) compared to the patients with low GV. With regards to the sepsis-related data, the severity of sepsis was high as evidenced by a high APACHE II score in both groups (27.5 ± 6.6 in the high GV group vs. 27.3 ± 6.6 in the low GV group, *p* = 0.70). The patients in the high GV group appeared to have a higher serum lactate level at 0 h (29.1 ± 27.4 vs. 24.5 ± 20.1 mg/dL, *p* = 0.05) and at 24 h (24.1 ± 23.6 vs. 19.9 ± 15.9, *p* = 0.10) than those in the low GV group. Moreover, the patients in the high GV group had a higher 30-day mortality rate compared to those in the low GV group (36.7% vs. 26.6%, *p* = 0.03). Given that DM is highly associated with a high GV, we investigated the specific role of DM in the association between GV and 30-day mortality. We used Kaplan–Meier analysis to test the correlation between GV and 30-day mortality, which showed that higher GV was associated with a higher risk of 30-day mortality (log-rank test, *p* = 0.018) (Fig. 2). The positive association between GV and 30-day mortality remained strong in the non-DM subgroup (log-rank test, *p* = 0.035), but was no longer present in the DM subgroup (log-rank test, *p* = 0.254) (Fig. 3). We also analyzed the GV between DM and non-DM groups in this study, and found that the patients with DM had higher GV including MAGE and CoV compared to those without DM. In this cohort, 30-day mortality was unaffected by DM (Fig. 4). Furthermore, in a multivariate Cox proportional hazard regression model adjusted for demographic, glycemia-associated and 30-day mortality-associated data (Additional file 1: Table S1), including age, sex, HbA1c, severe hypoglycemic episodes, cerebrovascular disease, hemoglobin and creatinine, APACHE II score (adjusted hazard ratio (aHR) 1.045, 95% confidence interval (CI) 1.013–1.078), level of lactate at 0 h (aHR 1.009, 95% CI 1.003–1.014), having a diagnosis of chronic airway disease (aHR 0.483, 95% CI 0.305–0.764), level of mean day 1 glucose (aHR 1.008, 95% CI 1.000–1.016), and high MAGE (aHR 1.607, 95% CI 1.008–2.563) were independently associated with 30-day mortality (Table 2). These findings showed that both mean day 1 glucose level and high MAGE were independently associated with 30-day mortality in patients with sepsis, and highlighted the importance of monitoring GV in critically ill patients.

**Table 2:** Cox proportional hazard regression analysis for 30-day mortality

Characteristics	Univariate		Multivariate	
	HR (95% CI)	*p* value	HR (95% CI)	*p* value
Age, per 1 year increment	1.003 (0.991–1.014)	0.63	1.005 (0.993–1.018)	0.41
Sex				
Female	1 [Reference]		1 [Reference]	
Male	1.113 (0.743–1.667)	0.60	1.292 (0.822–2.031)	0.27
HbA1c, per 1 increment	1.045 (0.935–1.167)	0.44	0.919 (0.803–1.051)	0.92
Cerebrovascular disease				
No	1 [Reference]		1 [Reference]	
Yes	0.602 (0.317–1.146)	0.12	0.502 (0.255–0.986)	0.05
Chronic pulmonary disease				
No	1 [Reference]		1 [Reference]	
Yes	0.506 (0.328–0.779)	< 0.01	0.483 (0.305–0.764)	< 0.01
APACHE II, per 1 increment	1.056 (1.027–1.086)	< 0.01	1.045 (1.013–1.078)	< 0.01
Lactate 0-h, per 1 mg/dL increment	1.010 (1.005–1.014)	< 0.01	1.009 (1.003–1.014)	< 0.01
Hemoglobin, per 1 g/dL increment	0.967 (0.818–2.672)	0.40	1.002 (09.25–1.085)	0.97
Creatinine, per 1 mg/dL increment	1.048 (0.993–1.105)	0.09	1.031 (0.954–1.115)	0.44
Hypoglycemia episode (< 40 mg/dL)				
No	1 [Reference]		1 [Reference]	
Yes	2.098 (0.293–15.009)	0.46	1.947 (0.245–15.47)	0.53
Mean day 1 glucose, per 1 mg/dL increment	1.004 (1.000–1.008)	0.04	1.008 (1.000–1.016)	0.04
Peak day 1 glucose, per 1 mg/dL increment	1.002 (1.000–1.004)	0.08	0.997 (0.992–1.001)	0.18
MAGE, cut-off point 65 mg/dL				
Low	1 [Reference]		1 [Reference]	
High	1.488 (1.068–2.072)	0.02	1.607 (1.008–2.563)	0.04

*HR* hazard ratio, *CI* confidence interval, *HbA1c* hemoglobin A1c, *APACHE II* acute physiology and chronic health evaluation II, *MAGE* mean amplitude of glycemic excursions

### Using CoV to assess GV

**Fig. 5:**
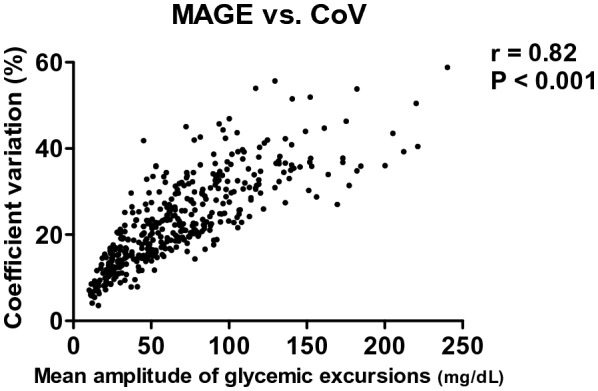
Correlations among the two indicators for glycemic variability. *MAGE* mean amplitude of glycemic excursions, *CoV* coefficient of variation

CoV is traditionally used to assess GV. Therefore, we tested the relationship between CoV and MAGE. Pearson correlation analysis showed a high positive correlation between these two measures for GV (MAGE vs. CoV, *r* = 0.82, *p* < 0.001) (Fig. 5). The multivariate Cox proportional hazard regression model, adjusted for the same variables used for MAGE, showed that a high GV (determined by CoV > 30%) was also independently associated with high 30-day mortality (aHR 2.593, 95% CI 1.494–4.499) (Table 3). Taken together, our findings suggested that high GV was common in the patients with sepsis, and that it was independently associated with 30-day mortality.

**Table 3:** Cox proportional hazard regression analysis for 30-day mortality

Characteristics	Univariate		Multivariate	
	HR (95% CI)	*p* value	HR (95% CI)	*p* value
Age, per 1 year increment	1.003 (0.991–1.014)	0.63	1.006 (0.993–1.019)	0.38
Sex				
Female	1 [Reference]		1 [Reference]	
Male	1.113 (0.743–1.667)	0.60	1.251 (0.798–1.959)	0.33
HbA1c, per 1 increment	1.045 (0.935–1.167)	0.44	0.926 (0.804–1.066)	0.28
Cerebrovascular disease				
No	1 [Reference]		1 [Reference]	
Yes	0.602 (0.317–1.146)	0.12	0.432 (0.218–0.856)	0.01
Chronic pulmonary disease				
No	1 [Reference]		1 [Reference]	
Yes	0.506 (0.328–0.779)	< 0.01	0.506 (0.320–0.800)	< 0.01
APACHE II, per 1 increment	1.056 (1.027–1.086)	< 0.01	1.040 (1.008–1.073)	< 0.01
Lactate 0-h, per 1 mg/dL increment	1.010 (1.005–1.014)	< 0.01	1.009 (1.003–1.015)	< 0.01
Hemoglobin, per 1 g/dL increment	0.967 (0.818–2.672)	0.40	0.983 (0.906–1.066)	0.68
Creatinine, per 1 mg/dL increment	1.048 (0.993–1.105)	0.09	1.028 (0.949–1.113)	0.50
Hypoglycemia episode (< 40 mg/dL)				
No	1 [Reference]		1 [Reference]	
Yes	2.098 (0.293–15.009)	0.46	1.660 (0.209–13.203)	0.63
Mean day 1 glucose, per 1 mg/dL increment	1.004 (1.000–1.008)	0.04	1.014 (1.005–1.024)	< 0.01
Peak day 1 glucose, per 1 mg/dL increment	1.002 (1.000–1.004)	0.08	0.993 (0.987–0.998)	0.01
CoV, cut-off point 30%				
Low	1 [Reference]		1 [Reference]	
High	1.683 (1.191–2.378)	< 0.01	2.593 (1.494–4.499)	< 0.01

*HR* hazard ratio, *CI* confidence interval, *HbA1c* hemoglobin A1c, *APACHE II* acute physiology and chronic health evaluation II, *CoV* coefficient of variation

## Discussion

In this study we investigated day 1 GV in patients with sepsis receiving protocol-based management of sepsis, and found that approximately 40% of the patients had high GV, defined as MAGE > 65 mg/dL. In addition, high day 1 GV was independently associated with 30-day mortality, and this relationship remained consistent when using CoV as a measure of GV. These findings highlight the critical role of GV in sepsis, and indicate the need for monitoring early GV in patients with sepsis.

GV refers to fluctuations in blood glucose level, which is a common stress response. However, there is currently no general consensus on its definition. The prevalence of GV in septic patients is unknown, which may partly be due to the lack of a standardized measurement of GV [20]. In addition, the timing of GV in patients with sepsis is also important, because it may have different clinical significance. GV in the early phase can be the result of a stress response, and late-phase GV may reflect the overall treatment responses. One strength of the present study is that all of the enrolled patients received regular glucose monitoring every 2 h, and an average of 10.6 ± 2.4 glucose measurements were taken within the initial 24 h of ICU admission. This early and intensive glucose monitoring enabled us to investigate the prevalence of high GV in the early phase of septic patients, and also its association with 30-day mortality. In line with our findings, Ali et al. reported that GV was associated with hospital mortality in patients with sepsis using all available glucose values for the entire hospitalization for sepsis in an administrative dataset [21]. In addition, a recent study focusing on the early phase of sepsis using all available glucose values within 48 h after admission via the emergency department, reported that CoV > 30% was associated with in-hospital mortality. However, due to the retrospective design and the difficulty of protocol-based intensive glucose monitoring in an emergency department, only 24.9% (1537/6165) of the patients had more than two blood glucose values within 48 h. In addition, the diagnosis of DM was limited due to the lack of HbA1c values in the patients without DM [18]. Taken together, these findings suggest the importance of GV in critically ill patients. Our findings further showed the prevalence of high GV in the early phase of sepsis through intensive glucose monitoring. It is therefore unsurprising that a high GV has been proposed to be incorporated into the severity score for critically ill patients [22].

The pathophysiological role of GV in critical illness is complex, and the association between DM and GV remains elusive. One recently published study measuring insulin sensitivity every 6 h for 72 h reported that insulin sensitivity was higher in non-survivors than in survivors [23]. This finding indicates that high insulin sensitivity may reflect a stress response in non-surviving septic patients, and that high insulin sensitivity may in turn lead to a high GV in these patients, as shown in the present study.

Previous studies and our data showed that high GV was associated with high mortality in non-diabetic critically ill patients [7, 24], whereas the association between high GV and patients with DM was less prominent than in those without DM (Fig. 3a, b). Silveira et al. retrospectively used glucose values from capillary blood during the overall ICU stay to determine MAGE in patients with sepsis, and reported that the patients with DM tended to have a higher GV than those without DM [25]. In the present study, we also found that the patients with DM had higher GV including MAGE and CoV than those without DM (Fig. 4a, b). Moreover, in our previous study, we also identified that a low glucose level (≤ 120 mg/dL) under glycemic control in patients with sepsis was associated with an increased risk of 14-day mortality in non-DM patients, but not in DM patients [26]. Therefore, septic patients with DM may have a higher tolerability to GV than septic patients without DM. In line with these findings, Krinsley et al. demonstrated that increased GV was independently associated with an increased risk of mortality among patients without DM. They also suggested that patients with DM may benefit from a higher glucose target range than those without DM [11]. The difference in tolerability to GV between patients with and without DM is complex, however it is likely that GV in patients with DM may reflect underlying variations in insulin secretion or sensitivity, whereas GV in patients without DM during sepsis may represent a survival response through interactions between insulin signaling pathways, particularly the GLUT-4 pathway, and activation of pro-inflammatory cascades during sepsis as proposed in so-called stress hyperglycemia [27, 28]. However, the association between GV and mortality may be due to an epiphenomenon of sepsis-associated dysglycemia instead of iatrogenic management; therefore, attempts to manipulate GV may not lead to improved outcomes.

In the present study, we used serial glucose data in a regular (every 2 h) glucose monitoring protocol to explore the critical role of GV in the early phase of sepsis. We found that making high-frequency glucose measurements was feasible and could be practically implemented as part of routine care in an ICU [11]. Advances in continuous glucose monitoring (CGM) technology will further enable intense surveillance for early GV in managing patients with sepsis. We suggest that more studies are needed to investigate the optimal glucose control strategy in CGM for patients with high GV [29].

There are several limitations to this study. First, this was a retrospective study; however, the protocol-based intensive glucose monitoring allowed us to accurately measure day 1 GV. Second, we excluded 65 patients without HbA1c data to avoid the potential misclassification of DM. However, the proportion of high GV in these 65 patients was 33.8% (22/65), and this would not have changed the magnitude of associations in the 452 patients. Third, we focused on day 1 glucose alone in the present study, given that the retrospective design did not allow us to precisely assess the GV beyond day 1. Fourth, the generalizability of the finding should be applied with caution given the high rates of male patients and patients with DM in this study which was conducted at a veteran’s hospital. Finally, a variety of treatments may influence blood sugar, including nutrition intake, glucose solution infusion and steroid administration. This retrospective analysis is limited by not including these factors because of the integrity and availability of the data.

## Conclusions

In conclusion, high day 1 GV was prevalent in the patients with sepsis, and it was independently associated with high 30-day mortality, particularly in the patients without DM. These findings highlight the crucial need of surveillance for early GV in patients with sepsis, such as with a CGM system. Additional studies are required to explore the mechanisms underlying GV and optimize glucose control.

## Supplementary information

**Additional file 1: Table S1.** Characteristics of the 452 patients with sepsis categorized by 30-day mortality.

## Data Availability

The datasets consulted during this study are available from the corresponding author on reasonable request.

## Abbreviations

GV: Glycemic variability
ICU: Intensive care unit
MAGE: Mean amplitude of glycemic excursions
CoV: Coefficient of variation
DM: Diabetes mellitus
aHR: Adjusted hazard ratio
CI: Confidence interval
APACHE II score: Acute physiology and chronic health evaluation II score
HbA1c: Glycated hemoglobin
SD: Standard deviation
CGM: Continuous glucose monitoring
