# Supplementary material for: Higher glycemic variability within the first day of ICU admission is associated with increased 30-day mortality in ICU patients with sepsis
Source: Ann Intensive Care. 2020 Feb 7;10:17. doi: 10.1186/s13613-020-0635-3 (PMC7007493; doi:10.1186/s13613-020-0635-3)
Supplement: Supplementary file 1 — Additional file 1: Table S1. Characteristics of the 452 patients with sepsis categorized by 30-day mortality. [file 13613_2020_635_MOESM1_ESM.docx]

| **Additional file 1: Table S1. Characteristics of the 452 patients with sepsis categorized by 30-day mortality** | | | | |
| --- | --- | --- | --- | --- |
|  | **All** | **Survivor** | **Non-survivor** | ***P* value** |
|  | **(N=452)** | **(N=312)** | **(N=140)** |  |
| **Basic and glycemia data** |  |  |  |  |
| Age (years) | 71.4±14.7 | 71.2±14.7 | 72.1±14.8 | 0.54 |
| Male % | 346 (76.7%) | 236 (75.9%) | 110 (78.6%) | 0.61 |
| BMI (kg) | 23.7±8.8 | 24±10.2 | 23±4.1 | 0.30 |
| HbA1c (%) | 6.3±1.4 | 6.2±1.4 | 6.4±1.5 | 0.45 |
| **Day 1 glucose metrics** | | | | |
| Mean glucose (mg/dL) | 164.1±41.7 | 161.6±38.0 | 169.8±48.6 | 0.08 |
| Peak glucose (mg/Dl) | 239.9±81.6 | 234.9±77.8 | 250.9±89.0 | 0.07 |
| Hypoglycemia (<40 mg/dL) | 2 (0.4%) | 1 (0.3%) | 1 (0.7%) | 0.52 |
| Glycemic variation |  |  |  |  |
| MAGE | 67±51.1 | 63.3±48.2 | 75.3±56.3 | 0.02 |
| CoV | 23.5±11.2 | 22.7±10.6 | 25.3±12.2 | 0.03 |
| **Comorbidities** |  |  |  |  |
| Diabetes mellitus | 158 (35%) | 108 (34.6%) | 50 (35.7%) | 0.91 |
| Congestive heart failure | 143 (31.6%) | 97 (31.1%) | 46 (32.9%) | 0.79 |
| Cerebrovascular disease | 50 (11.1%) | 40 (12.8%) | 10 (7.1%) | 0.10 |
| Chronic airway disease | 127 (28.1%) | 102 (32.7%) | 25 (17.9%) | <0.01 |
| Chronic renal disease | 53 (11.7%) | 36 (11.5%) | 17 (12.1%) | 0.88 |
| Malignancy | 108 (23.9%) | 70 (22.4%) | 38 (27.1%) | 0.29 |
| **Severity-associated variables** |  |  |  |  |
| APACHE II score | 27.4±6.6 | 26.5±6.4 | 29.5±6.6 | <0.01 |
| Lactate level, 0 h (mg/dl) | 26.5±23.6 | 23.7±22.1 | 32.8±25.7 | <0.01 |
| Lactate level, 24 h (mg/dl) | 21.7±19.6 | 18.6±13.8 | 27.8±26.6 | <0.01 |
| ScvO2, 0 h (%) | 74.7±11.4 | 74.3±10.6 | 75.7±13 | 0.26 |
| **Laboratory data** |  |  |  |  |
| Albumin (mg/dL) | 2.8±0.6 | 2.8±0.6 | 2.8±0.6 | 0.26 |
| Hemoglobin (g/dL) | 10.2±2.3 | 10.3±2.3 | 9.9±2.5 | 0.12 |
| Creatinine (mg/dL) | 2.1±2.2 | 1.9±2.3 | 2.4±2.1 | 0.04 |
| C-reactive protein (mg/dL) | 13.8±10.6 | 13.4±10.3 | 14.9±11.2 | 0.22 |
| Procalcitonin (ng/mL) | 17.4±34.9 | 17.1±32.9 | 17.9±39.2 | 0.84 |
| Data are presented as mean ± SD and N (%). GV, glycemic variability; MAGE, mean amplitude of glycemic excursions; CoV, coefficient of variation; DM, diabetes mellitus; BMI, body-mass index; HbA1c, hemoglobin A1c; APACHE II, Acute Physiology and Chronic Health Evaluation II; ScvO2, central venous oxygen saturation. | | | | |
